# Literacy Profiles in Twice-Exceptional Preadolescents with Intellectual Giftedness and Dyslexia

**DOI:** 10.3390/bs16061036

**Published:** 2026-06-21

**Authors:** Samuel Alonso Benito, Luz Florinda Pérez Sánchez, Ángeles Bueno Villaverde

**Affiliations:** Department of Education, Faculty of Education, Universidad Camilo José Cela, 28692 Madrid, Spain

**Keywords:** intellectual giftedness, dyslexia, twice-exceptionality, reading processes, writing processes, learning disability, underidentification

## Abstract

Research on twice-exceptional students, particularly those with co-occurring intellectual giftedness and dyslexia, remains limited and conceptually fragmented. This study examines the reading- and writing-related profiles of these students by comparing three groups: gifted students without dyslexia (G), gifted students with dyslexia (G-D), and dyslexic students without intellectual giftedness (D). The sample consisted of 133 Spanish-speaking primary school students (Grades 3–6). The results revealed a distinct and non-linear performance pattern. G-D students exhibited marked difficulties in lower-level literacy processes, including phonological and lexical processing, with a performance pattern closer to that of dyslexic peers. However, they showed relative strengths in higher-order language abilities, particularly text comprehension, oral comprehension, and written composition. The findings suggest a non-uniform profile of reading- and writing-related abilities in these students, characterized by weaknesses in several lower-level literacy processes and relative strengths in some higher-order language abilities. This pattern may contribute to the underidentification of these students across educational and clinical contexts. By providing empirical evidence from Spanish, a relatively underexplored orthographic context, this study contributes to current models of twice-exceptionality and highlights the need for more sensitive and staged identification procedures, as well as multidimensional assessment and intervention approaches that address both strengths and weaknesses.

## 1. Introduction

In recent years, increasing attention has been directed toward students who simultaneously exhibit intellectual giftedness and learning disabilities, a condition commonly referred to as twice-exceptionality. Among these profiles, the coexistence of giftedness and dyslexia represents a particularly complex and underexplored case, as advanced cognitive abilities may coexist with persistent deficits in literacy-related processes. This duality often leads to underidentification, as strengths and weaknesses can mask each other, hindering accurate diagnosis and appropriate educational support.

Intellectual giftedness is characterized by advanced cognitive functioning that enables individuals to outperform their peers across one or more domains, reflecting heightened efficiency in learning, problem-solving, and information processing (Calabrese et al., 2024; Gagné, 2020; Kuznetsova et al., 2024). In educational and clinical contexts, intellectual giftedness is typically identified through standardized cognitive assessments, with IQ thresholds commonly used as criteria, most often set at 130, corresponding to approximately the top 2% of the population (Baudry et al., 2026; François-Sévigny et al., 2025). However, giftedness does not always translate into optimal academic or developmental outcomes, as some students may present academic, social, or emotional difficulties that often remain underidentified (Baudry et al., 2026; Yiğit & Doğan, 2024). These issues highlight the complexity of the construct and underscore the need for more precise identification procedures and the implementation of differentiated educational strategies (Cross & Coleman, 2014; National Association for Gifted Children, 2019; Pérez & Freitas, 2016).

Within this context, twice-exceptionality (2e) refers to the coexistence of giftedness and a learning disability, resulting in uneven cognitive and academic profiles (McCoach et al., 2004). Although prevalence estimates vary, 2e is thought to affect approximately 1–5% of the school-aged population (McCoach et al., 2004; Nielsen, 2002). Research consistently highlights the heterogeneity of these profiles, with individuals often demonstrating advanced reasoning and metacognitive abilities alongside deficits in specific cognitive domains, such as working memory, processing speed, or domain-specific skills (Assouline et al., 2010; Maddocks, 2020; Toffalini et al., 2017). Importantly, the variability across types of learning disabilities complicates the interpretation of group-level findings and limits the generalizability of existing evidence (Kranz et al., 2024).

One of the most common learning disabilities is dyslexia, a neurobiological origin characterized by difficulties in reading, including accurate and fluent word recognition, decoding, and spelling, in individuals with adequate intelligence, motivation, and educational opportunities, and occurring in the absence of sensory, neurological, or primary intellectual deficits (Bonnetier, 2024; Eissa, 2010; Krafnick & Evans, 2019). From a cognitive perspective, dyslexia has been primarily associated with deficits in phonological processing, which hinder the representation and manipulation of speech sounds and, consequently, access to written language (Snowling et al., 2026). However, impairments in earlier stages of the reading system have also been identified, such as difficulties in encoding letter positions within words, which may lead to characteristic letter migration errors (e.g., reading “slime” as “smile” in English, or “calvo” as “clavo” in Spanish), highlighting the heterogeneity of cognitive profiles within the disorder (Kohnen et al., 2012). Epidemiologically, dyslexia has a substantial prevalence, generally estimated to affect between 5% and 13% of the population (American Psychiatric Association, 2022; Krafnick & Evans, 2019). Its high prevalence, together with its academic and socio-emotional consequences, highlights the importance of accurate identification and targeted intervention (Eissa, 2010).

The intersection of giftedness and dyslexia gives rise to a distinctive and complex profile in which cognitive strengths may partially compensate for core literacy deficits (Assouline et al., 2010). Gifted students often demonstrate advanced vocabulary, syntactic knowledge, and reading comprehension (Hoh, 2005; Reis et al., 2004), whereas individuals with dyslexia typically exhibit impairments in phonological awareness, decoding, and reading fluency (Snowling et al., 2020). In gifted students with dyslexia (G-D), these contrasting characteristics may coexist, leading to atypical performance patterns in which high-level language abilities obscure underlying deficits in foundational reading processes (Berninger & Abbott, 2013; van Viersen et al., 2017, 2016). This uneven pattern may complicate both clinical and educational identification, as neither the strengths nor the difficulties may be sufficiently salient to trigger assessment or intervention (Assouline et al., 2010; Kranz et al., 2024; Nicpon et al., 2011).

Despite growing interest in twice-exceptionality, research on gifted students with dyslexia remains limited and methodologically heterogeneous, hindering comparability across studies (Kranz et al., 2024). Such heterogeneity is also reflected in prevalence estimates, which vary according to how giftedness, dyslexia, and twice-exceptionality are defined, assessed, and identified across educational and clinical contexts. In educational settings, these students are frequently overlooked, as their cognitive strengths may mask underlying reading difficulties, resulting in profiles that do not clearly fit typical expectations (Snowling & Hulme, 2012). Such patterns may contribute to underidentification and may lead to inappropriate educational responses, with negative academic and socio-emotional consequences (Besnoy et al., 2015). Recent research further highlights the heterogeneity of literacy-related performance in this population, suggesting that the interaction between cognitive strengths and weaknesses can produce diverse outcomes, thereby complicating both identification and intervention processes (Alonso Benito et al., 2026). Early identification, combined with individualized educational interventions tailored to both strengths and weaknesses, may improve academic outcomes, enhance self-esteem, and reduce the risk of socio-emotional difficulties (Bermejo et al., 2013; Duric & Elgen, 2011; Valdés et al., 2013).

However, there is a lack of studies with substantial samples on G-D students that analyze different components of reading and writing separately, particularly in transparent orthographies such as Spanish. This distinction is important because, in educational and clinical identification contexts, reliance on global academic or literacy indicators may obscure uneven profiles in which word-level reading and spelling difficulties coexist with relatively better performance in comprehension or written expression. In the case of G-D students, broad indicators of reading or academic achievement may therefore be insufficient to detect these discrepancies. Therefore, the present study addresses this gap by examining the reading- and writing-related profiles of gifted students with dyslexia in comparison with gifted-only and dyslexic-only groups. These groups were selected because G-D students may not be clearly identified as either gifted or dyslexic when global academic or literacy indicators are considered. The use of standardized literacy measures also provides an age-based normative framework while keeping the focus of this study on the comparison between gifted-only, gifted dyslexic, and dyslexic profiles. Specifically, this study seeks to determine whether the interaction between intellectual giftedness and dyslexia-related deficits gives rise to a differentiated pattern of performance across literacy domains. It is hypothesized that students with giftedness and dyslexia will exhibit (a) weaknesses in lower-level processes, such as phonological and lexical processing, comparable to those observed in dyslexic peers; and (b) relative strengths in higher-order language abilities, such as text comprehension and written expression, with performance expected to be closer to that of their gifted peers than to that of dyslexic peers.

## 2. Materials and Methods

### 2.1. Participants

This study followed a retrospective observational design, using anonymized data obtained from psychological and educational assessment records. The sample was drawn from evaluations conducted between 2017 and 2025 at the Centro Psicológico y Educativo Huerta del Rey (Valladolid, Spain), a specialized center devoted to psychological and educational assessment. Therefore, this was a clinically referred sample rather than a school-based or population-based sample. However, referral to the center did not necessarily mean that participants had previously been identified as having a G-D profile; this classification was established retrospectively from the assessment records. Importantly, this sampling context provided relevant methodological strengths. Unlike group-administered assessments or school-based screening procedures, all participants were evaluated individually using a comprehensive psychoeducational assessment protocol in a setting specifically designed for this purpose. In addition, all assessments were conducted by the same highly experienced professional, a PhD in psychology specialized in child and adolescent neuropsychology. This procedure increased the consistency of the assessment process and reduced potential variability associated with differences in examiners, testing contexts, or administration conditions. Accordingly, although the clinically referred nature of the sample should be considered when interpreting the generalizability of the findings, it also ensured a high level of diagnostic consistency and depth of assessment. Participants were enrolled in grades 3 through 6 of primary education in the Spanish educational system and were aged between 8 and 12 years. They were all native Spanish speakers and came from various regions of Spain, predominantly from middle-to-high socioeconomic backgrounds. In all cases, informed consent was obtained from the families.

Participants were included in the dyslexia groups when the assessment records contained a clinical diagnosis of dyslexia established through a comprehensive psychoeducational evaluation. This diagnostic evaluation was not based exclusively on the PROLEC-R or PROESC tasks analyzed in the present study, but on a broader assessment battery comprising multiple cognitive, reading, writing, and psychoeducational measures. For the purposes of the present study, the Wechsler indices, PROLEC-R, and PROESC measures were selected from this broader assessment battery because they provided standardized indicators directly aligned with the study aims. The assessment records available for the present study did not indicate recorded comorbid neurodevelopmental or learning conditions that could provide an alternative explanation for the observed reading and writing difficulties. Intellectual giftedness was defined as a score of 130 or higher on the General Ability Index (GAI) derived from the Wechsler Intelligence Scale for Children (Wechsler, 2014). Although contemporary approaches to giftedness increasingly emphasize multidimensional identification procedures that go beyond IQ-based criteria, the present study operationalized intellectual giftedness using the GAI for research purposes rather than as a comprehensive diagnostic or educational identification model. This criterion was adopted because it provided an objective, standardized, and replicable basis for sample selection across retrospective assessment records. The GAI is considered a more appropriate indicator of intellectual ability in twice-exceptional populations, as it minimizes the influence of working memory and processing speed, domains in which these students often show relative weaknesses. In contrast, reliance on the Full Scale IQ (FSIQ) may underestimate their cognitive potential (Assouline et al., 2010; Maddocks, 2020; Toffalini et al., 2017).

The final sample consisted of 133 participants divided into three groups: students with intellectual giftedness without dyslexia (G; *n* = 55), students with intellectual giftedness and dyslexia (G-D; *n* = 39), and students with dyslexia without intellectual giftedness (D; *n* = 39). It should be noted that the D group was defined as dyslexic students without intellectual giftedness, rather than as a group with average intellectual ability. As shown in Table 1, this group had a mean GAI in the high-average range, and this characteristic should be considered when interpreting group comparisons.

### 2.2. Instruments

The assessment battery was selected to capture intellectual functioning, processing speed, and different components of reading and writing. Before conducting the analyses, measures were organized conceptually into the following domains: intellectual ability and processing speed, assessed with the Wechsler Intelligence Scale for Children (Wechsler, 2014); lower-level writing processes, assessed through syllable, word, and pseudoword dictation tasks from the PROESC: Writing Processes Assessment Battery (Cuetos et al., 2004); written expression, assessed through story and essay writing tasks from the PROESC; lower-level reading processes, assessed through letter naming, same–different discrimination, word reading, and pseudoword reading from the PROLEC-R: Reading Processes Assessment Battery—Revised (Cuetos et al., 2014); and higher-order reading and oral language abilities, assessed through grammatical structures, punctuation marks, sentence comprehension, text comprehension, and oral comprehension tasks from the PROLEC-R.

This organization was based on the cognitive demands of each task, distinguishing foundational processes related to letter identification, decoding, lexical access, and spelling accuracy from more complex syntactic, semantic, comprehension, and written expression processes. Although punctuation marks also involve fluent oral reading, this task was treated as a borderline higher-order measure because the PROLEC-R manual places it within syntactic processes and successful performance requires prosodic–syntactic integration (Cuetos et al., 2014). This organization reflects this study’s aim of examining whether G-D students show different patterns of performance across foundational literacy skills and higher-order language abilities.

Wechsler Intelligence Scale for Children (Wechsler, 2014):

- Intellectual level: The required indices were derived from the subtests to compute the General Ability Index (GAI), which provides an estimate of overall intellectual ability while minimizing the influence of working memory and processing speed. This measure is particularly recommended for the assessment of students with learning disorders (Assouline et al., 2010; Kalbfleisch, 2014; Toffalini et al., 2017). The reliability coefficient reported in the test manual exceeds 0.92.
- Processing speed: Processing speed was assessed using the relevant subtests of the same instrument. This index reflects the efficiency with which individuals can perform simple cognitive tasks under time constraints, involving visual scanning, attention, and graphomotor speed. The reliability coefficient reported in the test manual is 0.88.

PROESC: Writing Processes Assessment Battery (Cuetos et al., 2004). The reliability coefficient reported in the manual for all tests is 0.82:

- Syllable dictation: Children are asked to write 25 syllables representing the main syllabic structures. This task provides information about basic phoneme–grapheme conversion processes.
- Word dictation: This task includes two lists of 25 words. List A contains words with arbitrary spelling, whereas List B includes words governed by spelling rules. The latter is used to obtain the ruled spelling score.
- Pseudoword dictation: Children write 25 invented words. A total score is obtained, and an additional score is calculated from the last 15 pseudowords, which follow orthographic rules.
- Writing a story: Children are asked to write a story based on a prompt. The task is scored according to content and coherence-related aspects.
- Writing an essay: Children write an essay about a familiar animal. Performance is evaluated according to content and presentation-related aspects.

PROLEC-R: Reading Processes Assessment Battery—Revised (Cuetos et al., 2014):

- Letter name or sound: This task examines knowledge of letters and their corresponding sounds. Completion time is also recorded as an indicator of automaticity in letter recognition and naming. The reliability coefficient reported in the test manual is 0.91.
- Same–different: Children are asked to determine whether pairs of written stimuli are the same or different, which provides information about their ability to segment and identify the letters within words. The reliability coefficient reported in the test manual is 0.76.
- Word reading: This task involves reading real words aloud. Both accuracy and reading time are recorded, providing information about lexical reading processes. The reliability coefficient reported in the test manual is 0.95.
- Pseudoword reading: Children are asked to read unfamiliar or non-existent words aloud. This task provides information about grapheme–phoneme conversion and sublexical reading processes. The reliability coefficient reported in the test manual is 0.86.
- Grammatical structures: This task evaluates syntactic processing through sentences with different grammatical structures. The reliability coefficient reported in the test manual is 0.88.
- Punctuation marks: Children read a short text aloud while respecting the intonation indicated by punctuation marks. This task provides information about the use of punctuation during oral reading. The reliability coefficient reported in the test manual is 0.89.
- Sentence comprehension: This task examines the child’s ability to understand different types of sentences and respond according to their meaning. The reliability coefficient reported in the test manual is 0.85.
- Text comprehension: Children read short narrative and expository texts and answer comprehension questions about their content. This task provides information about their ability to extract and integrate information from written texts. The reliability coefficient reported in the test manual is 0.87.
- Oral comprehension: In this task, the examiner reads two expository texts aloud, and the child then answers comprehension questions. The reliability coefficient reported in the test manual is 0.81.

### 2.3. Data Analysis

Statistical analyses were conducted using SPSS version 25 and G*Power version 3.1. The analytical procedure comprised an initial descriptive phase, followed by an inferential phase.

Prior to group comparisons, normality was assessed using the Shapiro–Wilk test to determine the most appropriate statistical approach. For normally distributed variables, homogeneity of variance was assessed using Levene’s test. Standard one-way ANOVA was used when this assumption was met, whereas Welch’s ANOVA was applied when it was not met (McKnight & Najab, 2010). For variables that did not meet the normality assumption, the non-parametric Kruskal–Wallis test was applied (Ostertagova et al., 2014).

Effect sizes were calculated to estimate the magnitude of the observed differences. When significant differences were identified, post hoc pairwise comparisons were conducted. Bonferroni-adjusted post hoc comparisons were used after standard ANOVA, whereas Tamhane’s T2 was used after Welch’s ANOVA. For Kruskal–Wallis tests, pairwise comparisons of mean ranks were conducted with Bonferroni-adjusted significance values. Cohen’s d was used for parametric pairwise comparisons (Cohen, 1988), whereas effect size r was calculated for non-parametric pairwise comparisons as r = |Z|/√N. Statistical power (1 − β) was also calculated and the significance level was set at *p* < 0.05 (Muller & Benignus, 1992). Given the exploratory nature of this study and the difficulty of recruiting students with this twice-exceptional profile, *p*-values were not adjusted across the full set of outcome variables. Accordingly, the results were interpreted cautiously, with emphasis placed on the consistency of patterns across related measures, effect sizes, statistical power, and theoretical coherence rather than on isolated *p*-values alone.

## 3. Results

The sample showed a higher proportion of males than females. A chi-square test indicated that there were no statistically significant differences in gender distribution across groups, χ^2^(2) = 0.56, *p* = 0.757, suggesting that the male predominance was consistent among the G, G-D, and D groups. Similarly, no statistically significant differences were found in age in months, H(2) = 0.20, *p* = 0.905, or in the distribution of participants across academic grades, indicating that the groups were comparable in age and grade level.

Table 2 and Table 3 report the main results of the analyses, including statistically significant outcomes, effect sizes, and statistical power. Table 2 provides descriptive statistics and group comparisons for processing speed and writing-related measures obtained from the PROESC battery, along with the type of test applied and associated statistical indicators. Table 3 presents the corresponding information for reading-related measures derived from the PROLEC-R battery. Non-significant results indicate a lack of statistically significant differences between groups at the established alpha level; however, they should not be interpreted as evidence of equivalence between groups. Therefore, nonsignificant findings were interpreted cautiously and considered together with descriptive patterns, effect sizes, and statistical power. Table A1 and Table A2 present the normative reference values used to contextualize the PROESC and PROLEC-R scores, respectively, as provided by the test manuals.

In processing speed, groups G and G-D obtained higher mean scores than group D, whereas no statistically significant differences were observed between G and G-D. A statistically significant difference was found between G and D (*p* < 0.001), suggesting that the G-D group showed a processing speed profile closer to the gifted group than to the dyslexic group.

Lower-level writing processes showed a pattern broadly consistent with the first part of the hypothesis. In syllable dictation, the G-D and D groups obtained close mean scores, with no statistically significant difference between them, whereas both groups differed significantly from the G group (*p* < 0.001). A similar pattern was observed in several spelling-related measures, including arbitrary spelling word dictation, ruled spelling word dictation, total pseudoword dictation, and pseudoword dictation with orthographic rules. Although the G-D group obtained somewhat higher mean scores than the D group in these tasks, the differences between these two groups were not statistically significant, while both groups showed lower performance than the G group.

Lower-level reading processes showed a broadly comparable pattern, although not uniformly across all measures. In same–different discrimination and word reading, the G-D group performed below the G group and close to the D group. In pseudoword reading, however, the G-D group obtained a lower mean score than the D group, indicating a particularly pronounced weakness in phonological decoding. A similar non-intermediate pattern was observed in some other lower-level or borderline measures, such as pseudoword dictation and punctuation marks, where the G-D group performed close to the D group rather than clearly between the G and D groups. Although the G-D vs. D comparisons were not statistically significant, these descriptive patterns suggest that some foundational literacy processes may represent specific areas of vulnerability in G-D students. These findings partially support the hypothesis that G-D students show weaknesses in foundational literacy processes, particularly in phonological, lexical, and spelling-related tasks.

Higher-order writing processes showed a different pattern from lower-level writing tasks. In written expression tasks, including writing a story and writing an essay, the G and G-D groups obtained higher mean scores than the D group, and no statistically significant differences were observed between G and G-D. These findings suggest that the G-D group showed relatively stronger performance in written expression than in lower-level spelling and dictation tasks.

Higher-order reading and oral language processes also showed a relatively preserved pattern of performance. In grammatical structures, the gifted groups obtained higher mean scores than the D group, although the comparison between G and G-D was not statistically significant. In sentence comprehension, no statistically significant differences were observed between groups. However, group means were close to the maximum possible score of 16 across all three groups, suggesting a ceiling effect that may have limited the sensitivity of this task to detect between-group differences. In text comprehension and oral comprehension, the G-D group obtained the highest mean scores, although the pairwise comparison with the G group was not statistically significant. In both measures, G and G-D differed significantly from the D group. These findings partially support the second part of the hypothesis, suggesting relatively stronger performance in higher-order language abilities among G-D students than in lower-level literacy processes. However, given the nonsignificant pairwise comparisons, these results should be interpreted as a pattern of relative strengths rather than evidence of equivalence with gifted peers.

## 4. Discussion

The present study suggests a non-uniform profile of literacy-related abilities in G-D students. Although these students showed an intermediate pattern of performance between gifted and dyslexic peers in several measures, this pattern was not consistent across domains. Specifically, G-D students exhibited clear weaknesses in lower-level processes, particularly phonological and lexical processing, and in some foundational measures, their performance was at or near the level of dyslexic peers rather than clearly intermediate. This was especially evident in pseudoword reading, where the G-D group obtained a lower mean score than the D group, as well as in other lower-level or borderline measures such as pseudoword dictation and punctuation marks, where their performance was close to that of the D group. In contrast, G-D students showed relative strengths in higher-order language abilities. Notably, in higher-order tasks such as narrative writing, text comprehension, and oral comprehension, the G-D group showed a more preserved pattern of performance than in lower-level literacy tasks, with mean scores closer to those of the gifted group than those observed in phonological and lexical measures. However, these patterns should not be interpreted as evidence of equivalence between G-D and G students. Similarly, the absence of significant group differences in sentence comprehension should be interpreted cautiously, as all three groups obtained mean scores close to the maximum possible score, suggesting a ceiling effect and limited sensitivity of this task to detect subtle between-group differences. The nonsignificant differences between the G and G-D groups in letter name or sound and grammatical structures should also be interpreted cautiously. In both tasks, the G-D group showed mean scores between those of the G and D groups, suggesting an intermediate descriptive pattern despite the absence of statistically significant pairwise differences. This may reflect the relatively basic and consolidated nature of letter identification in students from 3rd to 6th Grade, and the relative preservation of syntactic processing in G-D students. Taken together, these findings suggest that the coexistence of giftedness and dyslexia does not simply result in an intermediate profile, but rather in a complex pattern of coexisting strengths and weaknesses. Importantly, this uneven profile highlights the need to examine the different components involved in reading and writing separately, rather than relying exclusively on global indicators of academic or literacy performance.

These findings can be interpreted within a framework that distinguishes between lower-level and higher-level language processes. The results suggest that phonological and lexical difficulties associated with dyslexia may be less influenced by intellectual ability, whereas higher-order language skills may be supported by cognitive strengths in gifted students. This interpretation is consistent with the move away from IQ–achievement discrepancy models of dyslexia, as Stanovich’s work challenged the use of IQ as a gatekeeper for identifying dyslexia, and poor readers have been shown to present similar phonological difficulties irrespective of IQ level (Snowling et al., 2020; Stanovich, 1996). Evidence from studies on gifted children with dyslexia supports this interpretation, showing no clear indication that higher cognitive abilities directly compensate for underlying phonological impairments (van Viersen et al., 2016). At the same time, more recent research suggests that cognitive strengths may operate at a different level of processing. Rather than remediating the core deficit itself, these strengths, particularly in language-related domains, may be associated with performance patterns consistent with compensatory strategies in more complex tasks (van Viersen et al., 2019). This distinction aligns with the differentiation between proximal (word-level) and distal (language-level) processes in reading models. As a result, students with and without intellectual giftedness may experience similar difficulties in the underlying processes involved in word reading and writing while differing in their ability to cope with these difficulties at higher levels of language processing.

These findings are consistent with previous research on intellectually gifted students with dyslexia, such as the study by Berninger and Abbott (2013). These authors found that students with dyslexia and superior verbal reasoning outperformed those with average verbal reasoning in higher-level literacy skills, including reading, spelling, and morphological and syntactic processing. However, no differences were observed in core processes associated with dyslexia, such as phonological and orthographic word-form storage and processing, or in related working memory components. One possible explanation for these patterns lies in differences in how intellectual ability is operationalized. In the study by Berninger and Abbott (2013), group classification was based on verbal reasoning scores, whereas the present study employed the General Ability Index (GAI), which has been proposed as a more appropriate measure of intellectual functioning in individuals with learning disabilities (Toffalini et al., 2017). In contrast to the Full Scale IQ (FSIQ), the GAI excludes working memory and processing speed components, which are often impaired in this population and may therefore lead to an underestimation of their cognitive abilities. More broadly, the lack of consensus regarding identification criteria for twice-exceptional students may contribute to inconsistencies across studies (Assouline et al., 2010; Maddocks, 2018; Nicpon et al., 2011). The use of different cognitive indicators and cut-off scores may substantially influence both sample composition and observed results. In this context, the present study contributes to the literature by adopting an approach that aims to better capture the intellectual potential of students with learning disabilities.

In interpreting this pattern, it is important to distinguish between compensation and masking. Compensation refers to the use of cognitive or language-related strengths, or specific strategies, to reduce the functional impact of an underlying deficit and improve performance in certain literacy tasks (van Viersen et al., 2019). Masking, in contrast, refers to a situation in which such strengths make the literacy difficulty less visible without eliminating the underlying dyslexia-related deficit (Maddocks, 2018; van Viersen et al., 2016). The present findings appear more consistent with masking than with a strong compensatory account. Although the G-D group showed relative strengths in higher-order language tasks, their performance remained weak in several foundational literacy measures, particularly those involving phonological decoding, lexical access, and spelling-related processes. Thus, the observed strengths may have made the profile less uniformly impaired, but they did not appear to eliminate the lower-level difficulties associated with dyslexia, consistent with previous evidence indicating that giftedness-related strengths do not necessarily compensate for core dyslexia-related deficits (Berninger & Abbott, 2013; van Viersen et al., 2015).

The relatively stronger performance of the G-D group in text comprehension and oral comprehension may reflect the different demands of these tasks. Unlike word reading, pseudoword reading, or spelling, comprehension tasks rely more strongly on broader language resources, such as verbal reasoning, vocabulary, background knowledge, inferential processing, and listening comprehension (Snowling et al., 2026). This pattern is consistent with previous evidence suggesting that oral-language and reasoning strengths may support performance in higher-order literacy tasks, whereas word-level reading, fluency, and spelling weaknesses may remain evident in G-D students (Kranz et al., 2024; Maddocks, 2020). Therefore, these findings should be interpreted as relative strengths within an uneven profile, rather than as evidence that the underlying dyslexia-related difficulties were fully compensated for.

Consistent with this interpretation, previous research by van Viersen et al. (2015, 2016) shows a similar pattern to that observed in the present study, with G-D students often showing mean scores above those of D students but below those of G students across several reading and spelling measures. These findings partially support the notion of an intermediate performance profile in several lower-level literacy measures, while also indicating that the G-D profile is not uniform across domains. Importantly, this profile may be interpreted as consistent with masking processes, whereby relatively strong cognitive and language abilities may contribute to literacy performance that appears less impaired in some domains, despite underlying difficulties (van Viersen et al., 2016). In this sense, their performance may not fully reflect their cognitive potential, nor the severity of their underlying difficulties. While some authors have suggested that gifted students with learning disabilities may rely on compensatory strategies to support their academic performance (Nicpon et al., 2011), empirical evidence indicates that such strengths do not necessarily remediate the core deficits associated with dyslexia (van Viersen et al., 2015).

In contrast, G-D students appear to present a specific pattern of strengths and weaknesses characterized by the coexistence of risk and protective factors, which may facilitate relatively higher levels of performance in certain domains without eliminating their underlying difficulties. As a result, these students may continue to experience persistent challenges, particularly in tasks involving phonological and lexical processing. In the present study, this was especially evident in pseudoword reading, where the G-D group obtained the lowest mean score. This finding suggests that phonological decoding may remain particularly vulnerable in gifted students with dyslexia, even when other language-related abilities are relatively preserved, and is consistent with evidence indicating that giftedness-related strengths do not necessarily compensate for dyslexia-related deficits (van Viersen et al., 2015). More broadly, the fact that G-D students performed at or near the level of the D group on several foundational literacy measures suggests that intellectual giftedness may not exert the same protective influence across all components of reading and writing, particularly when tasks rely heavily on phonological decoding, lexical access, or automatized fluency. This interpretation is consistent with previous evidence that giftedness-related strengths do not necessarily compensate for core dyslexia-related deficits (Berninger & Abbott, 2013; van Viersen et al., 2015).

This complex profile may contribute to the challenges associated with their identification, as similar students may not exhibit sufficiently low academic performance, nor clearly stand out as high achievers, to warrant referral for diagnostic assessment, despite experiencing significant discrepancies between their potential and actual achievement. Although the present sample was clinically referred, referral did not necessarily mean that the G-D profile had been previously recognized; rather, this profile was identified through the comprehensive assessment process. Therefore, the findings do not estimate the prevalence of hidden G-D students in the broader school population, but they do illustrate a pattern of strengths and weaknesses that may help explain why similar profiles can remain underidentified or misunderstood in educational contexts.

When comparing findings across studies, it is essential to consider the role of orthographic transparency, as differences between writing systems can substantially influence literacy development and performance. Orthographic transparency refers to the consistency of grapheme–phoneme correspondences, which varies across languages and affects the cognitive processes involved in reading and writing (van Viersen et al., 2017). In this regard, languages with high transparency, such as Spanish, rely heavily on regular grapheme–phoneme mappings, which may shape both the manifestation of dyslexia and the strategies used to cope with reading difficulties (Ferreres & López, 2014). Consequently, direct comparisons between studies conducted in languages with differing levels of orthographic transparency should be interpreted with caution. The present study contributes to the literature by examining these phenomena in Spanish, a relatively transparent orthography that has been underrepresented in research on twice-exceptionality. This is particularly relevant, as the patterns observed in this context may differ from those reported in studies conducted in more opaque languages, where reading processes and compensatory mechanisms may operate differently. Thus, whereas the uneven profile characterized by lower-level literacy weaknesses and relatively stronger higher-order language abilities may be relevant across orthographies, the specific manifestation of reading and writing difficulties may depend on the transparency of the writing system.

These findings have important implications for both educational and clinical practice, particularly in the identification and support of G-D students. The results highlight the need to consider both strengths and weaknesses within a unified framework, as these students may simultaneously demonstrate high-level abilities and persistent underlying difficulties. Therefore, the GAI should not be interpreted as a sufficient standalone criterion for characterizing G-D students. Although it may help reveal intellectual potential that could be underestimated by literacy difficulties or by FSIQ scores, it does not identify the specific literacy processes in which difficulties persist. For this reason, attention to sub-profiles remains necessary to interpret intellectual potential alongside the student’s specific pattern of strengths and weaknesses in reading and writing. In practical terms, professionals should be attentive to profiles in which strong oral comprehension, text comprehension, reasoning, or vocabulary coexist with persistent difficulties in reading fluency, pseudoword decoding, spelling, or written accuracy. Such higher-order strengths may make literacy difficulties less visible when only global academic or literacy indicators are considered. Therefore, an initial line of identification should involve detecting uneven performance across domains, followed by a comprehensive diagnostic and psychoeducational assessment in which these characteristics can be evaluated separately. In this regard, a dually differentiated educational approach is required, combining enrichment in areas of strength with targeted support in areas of difficulty. Failure to adequately identify and support these students may result in negative consequences for their academic development, motivation, and emotional well-being. Conversely, appropriate recognition and intervention can help them to reach their full potential, ensuring a more equitable and responsive educational experience.

### Study Limitations and Future Research Directions

Although the sample size should be considered relatively substantial given the difficulty of identifying students with this twice-exceptional profile, the sample was drawn from a specialized assessment center and participants predominantly came from middle-to-high socioeconomic backgrounds. Therefore, as this was a clinically referred sample rather than a school-based or population-based sample, caution is warranted when generalizing the findings to broader educational populations or to students from different socioeconomic backgrounds. In addition, the D group showed a mean GAI in the high-average range and should therefore be interpreted as a group of dyslexic students without intellectual giftedness, rather than as a representative average-IQ dyslexic group. This characteristic may have reduced the magnitude of differences between the G-D and D groups. Although referral to the center did not necessarily imply that the G-D profile had been previously recognized, the present design does not allow the prevalence of hidden or underidentified G-D students in the broader school population to be estimated. Group classification was not based solely on the PROLEC-R or PROESC tasks analyzed in the present study; these measures were selected from a broader psychoeducational assessment battery for research purposes because they were directly aligned with the aims of this study. At the same time, the consistency and depth of the assessment procedure represent important methodological strengths, as all evaluations were conducted in the same specialized center, using a consistent assessment protocol, and carried out by the same highly experienced professional.

Second, the use of a comprehensive assessment battery represents a relevant strength of this study, as it allowed the complex profiles of these students to be examined across multiple reading, writing, and psychoeducational domains. However, such an in-depth assessment requires considerable time and resources, which may make its direct implementation more challenging in broader or school-based contexts. Additionally, the sample consisted exclusively of native Spanish speakers. Given the high orthographic transparency of Spanish, the profiles and performance patterns observed may differ from those found in more opaque languages, such as English. Despite these considerations, this study provides relevant evidence on an under-researched population using a comprehensive and methodologically rigorous and consistent approach.

Future research should continue to examine these profiles in larger and more diverse samples, including school-based and cross-linguistic contexts. Longitudinal designs would be especially valuable to examine developmental trajectories and compensatory mechanisms over time. Furthermore, incorporating additional cognitive and socio-emotional variables would contribute to a more comprehensive understanding of this population and to the refinement of identification and intervention models.

## 5. Conclusions

Research on 2e students remains limited, particularly in the case of those with intellectual giftedness and dyslexia. The present study contributes to this field by providing a detailed analysis of their literacy-related profile, highlighting the coexistence of significant difficulties in lower-level processes, such as phonological and lexical processing, alongside relative strengths in higher-order language abilities. The findings support the existence of a differentiated and uneven profile in G-D students, characterized by a partially intermediate but non-uniform pattern of performance between gifted and dyslexic peers. However, this intermediate pattern was not uniform across domains, as G-D students showed more pronounced weaknesses in lower-level literacy processes and comparatively stronger performance in higher-order language abilities. Although the present clinically referred sample cannot demonstrate underidentification directly, the observed pattern is consistent with the idea that similar G-D profiles may be difficult to recognize in broader educational contexts, particularly when stronger language-related abilities and high intellectual potential make reading and writing difficulties less apparent on global indicators of academic or literacy performance. This may occur despite meaningful discrepancies between students’ cognitive potential and academic performance.

From an educational perspective, these results underscore the need for more sensitive and staged identification procedures that consider both strengths and weaknesses within a unified framework. In particular, the findings highlight the importance of analyzing the different components involved in reading and writing separately, rather than basing identification exclusively on global measures of literacy or academic performance. Such an approach may help detect uneven profiles in which higher-order language abilities mask persistent difficulties in lower-level reading and writing processes. Early detection and the implementation of dually differentiated educational approaches, combining cognitive enrichment with targeted support in literacy-related skills, are essential to ensure that these students receive an appropriate and equitable educational response. Overall, this study advances current understanding of twice-exceptionality by providing empirical evidence from a relatively underexplored linguistic context and reinforces the importance of adopting multidimensional approaches to assessment and educational intervention in this population.

## Figures and Tables

**Table 1:** Descriptive Analysis: Sample Size, Percentage of Males/Females, Grade Level, Age in months and IQ Score by Group.

				Grade Level				Age in Months			IQ	
Group	*n*	% Male	% Female	3rd	4th	5th	6th	M	SD	Range	M	SD
Gifted (G)	55	61.82%	38.18%	14	9	20	12	120.73	12.98	98–146	138.84	7.443
Gifted Dyslexic (G-D)	39	64.10%	35.90%	10	12	7	10	120.69	15.33	98–150	136.13	6.622
Dyslexic (D)	39	69.23%	30.77%	11	11	8	9	121.67	13.55	97–143	115.56	8.958
Total	133	64.67%	35.34%	35	32	35	31	121.00	13.72	97–150	131.22	12.732

Note. M = mean; SD = standard deviation.

**Table 2:** Descriptive Statistics and Group Comparisons for Processing Speed and PROESC Measures.

	Gifted (G)		Gifted Dyslexic (G-D)		Dyslexic (D)				Gifted vs. Dyslexic			Gifted vs. Gifted Dyslexic			Gifted Dyslexic vs. Dyslexic		
	M	SD	M	SD	M	SD	Test	Omnibus Test	*p*-Value	Effect Size	1 − β	*p*-Value	Effect Size	1 − β	*p*-Value	Effect Size	1 − β
Processing speed	114.96	11.31	109.21	12.29	103.82	13.80	P	F(2, 130) = 9.38, *p* < 0.001, η^2^ = 0.13	<0.001 ***	0.903	0.867	0.063	0.491	-	0.218	0.412	-
Syllable dictation	23.74	1.19	22.56	1.67	22.69	1.43	NP	H(2) = 18.02, *p* < 0.001, η^2^H = 0.15	<0.001 ***	0.404	0.597	<0.001 ***	0.423	0.635	1.000	0.019	-
Word dictation—arbitrary spelling	21.38	3.56	18.29	5.17	16.14	4.49	NP	H(2) = 24.33, *p* < 0.001, η^2^H = 0.21	<0.001***	0.557	0.948	0.017 *	0.318	0.819	0.139	0.240	-
Word dictation—ruled spelling	22.29	3.54	19.97	3.80	18.17	3.71	NP	H(2) = 21.99, *p* < 0.001, η^2^H = 0.19	<0.001 ***	0.528	0.818	0.018 *	0.316	0.708	0.235	0.212	-
Pseudoword dictation—total score	20.74	3.12	19.48	2.50	18.94	2.60	NP	H(2) = 17.71, *p* < 0.001, η^2^H = 0.15	<0.001 ***	0.464	0.119	0.017 *	0.319	0.386	0.698	0.145	-
Pseudoword dictation—orthographic rules	11.57	2.63	10.64	1.83	10.23	2.07	NP	H(2) = 14.77, *p* < 0.001, η^2^H = 0.12	<0.001 ***	0.405	0.222	0.011 *	0.335	0.280	1.000	0.069	-
Writing a story	5.74	2.25	4.72	2.26	3.82	1.95	P	F(2, 130) = 7.57, *p* < 0.001, η^2^ = 0.12	0.010 **	0.908	0.770	0.165	0.455	-	0.267	0.425	-
Writing an essay	4.65	2.31	3.79	1.58	2.83	1.18	NP	H(2) = 13.36, *p* = 0.001, η^2^H = 0.11	<0.001 ***	0.428	0.795	0.408	0.171	-	0.113	0.262	-

Note. P = parametric test, including one-way ANOVA or Welch’s ANOVA when homogeneity of variance was not met; NP = non-parametric Kruskal–Wallis test. Effect sizes are reported as Cohen’s d for parametric comparisons and r for non-parametric comparisons. Pairwise *p*-values and effect sizes are reported for all comparisons; 1 − β = statistical power for significant post hoc comparisons. *p*-values are unadjusted and should be interpreted as exploratory. * *p* < 0.05; ** *p* < 0.01; *** *p* < 0.001.

**Table 3:** Descriptive Statistics and Group Comparisons for PROLEC-R Measures.

	Gifted (G)		Gifted Dyslexic (G-D)		Dyslexic (D)				Gifted vs. Dyslexic			Gifted vs. Gifted Dyslexic			Gifted Dyslexic vs. Dyslexic		
	M	SD	M	SD	M	SD	Test	Omnibus Test	*p*-Value	Effect Size	1 − β	*p*-Value	Effect Size	1 − β	*p*-Value	Effect Size	1 − β
Letter name or sound	166.90	42.51	151.54	38.80	144.53	36.63	NP	H(2) = 6.01, *p* = 0.050, η^2^H = 0.05	0.044 *	0.319	0.327	0.593	0.174	-	0.839	0.145	-
Same–different	32.93	8.22	23.81	9.70	24.17	10.26	NP	H(2) = 18.15, *p* < 0.001, η^2^H = 0.20	<0.001 ***	0.477	0.619	<0.001 ***	0.488	0.668	1.000	0.005	-
Word reading	158.93	42.85	110.62	48.39	109.47	38.51	P	F(2, 130) = 12.27, *p* < 0.001, η^2^ = 0.23	<0.001 ***	1.217	0.917	<0.001 ***	1.063	0.747	1.000	0.025	-
Pseudoword reading	83.48	26.39	49.58	18.44	58.31	14.48	P	Welch’s F(2, 50.93) = 15.67, *p* < 0.001	<0.001 ***	1.183	0.889	<0.001 ***	1.498	0.986	0.158	0.535	-
Grammatical structures	15.17	1.14	14.42	1.50	13.60	2.11	NP	H(2) = 12.78, *p* = 0.002, η^2^H = 0.13	<0.001 ***	0.463	0.641	0.125	0.275	-	0.478	0.188	-
Punctuation marks	26.59	4.82	20.81	7.81	20.52	5.52	P	F(2, 130) = 9.20, *p* < 0.001, η^2^ = 0.19	<0.001 ***	1.171	0.880	<0.001 ***	0.927	0.574	1.000	0.035	-
Sentence comprehension	15.90	0.31	15.69	0.47	15.60	0.62	NP	H(2) = 5.08, *p* = 0.079, η^2^H = 0.04	0.101	0.277	-	0.270	0.229	-	1.000	0.048	-
Text comprehension	14.34	1.45	14.62	1.36	13.03	2.16	NP	H(2) = 10.89, *p* = 0.004, η^2^H = 0.11	0.041 *	0.324	0.829	1.000	0.097	-	0.006 **	0.420	0.947
Oral comprehension	6.14	1.55	6.31	1.35	5.10	1.27	NP	H(2) = 12.20, *p* = 0.002, η^2^H = 0.12	0.014 *	0.368	0.696	1.000	0.055	-	0.005 **	0.422	0.780

Note. P = parametric test, including one-way ANOVA or Welch’s ANOVA when homogeneity of variance was not met; NP = non-parametric Kruskal–Wallis test. Effect sizes are reported as Cohen’s d for parametric comparisons and r for non-parametric comparisons. Pairwise *p*-values and effect sizes are reported for all comparisons; 1 − β = statistical power for significant post hoc comparisons. *p*-values are unadjusted and should be interpreted as exploratory. * *p* < 0.05; ** *p* < 0.01; *** *p* < 0.001.

## Data Availability

Data is contained within the article. The original contributions presented in this study are included in the article. Further inquiries can be directed to the corresponding author.

## Appendix A. Supporting Data

**Table A1:** Age-based normative reference values for PROESC measures from 3rd to 6th Grade.

	Grade Level							
PROESC	3rd		4th		5th		6th	
Measure	M	SD	M	SD	M	SD	M	SD
Syllable dictation	23.80	1.20	24.13	1.44	24.04	1.12	23.86	1.39
Word dictation—arbitrary spelling	17.88	3.87	18.51	3.86	20.46	3.07	21.59	2.93
Word dictation—ruled spelling	19.66	2.78	20.49	2.79	21.56	2.56	22.60	2.35
Pseudoword dictation—total score	22.87	2.14	22.67	2.20	23.73	1.82	23.90	1.41
Pseudoword dictation—orthographic rules	11.70	1.92	12.32	2.00	12.42	1.95	13.02	1.94
Writing a story	3.80	1.96	4.13	1.63	4.78	1.78	5.36	1.66
Writing an essay	1.90	1.43	2.12	1.72	3.39	2.37	3.82	2.01

Note. M = mean; SD = standard deviation. Normative values are taken from the PROESC manual for Spanish primary school students from 3rd to 6th Grade.

**Table A2:** Age-based normative reference values for PROLEC-R accuracy scores from 3rd to 6th Grade.

	Grade Level							
PROLEC-R	3rd		4th		5th		6th	
Measure	M	SD	M	SD	M	SD	M	SD
Letter name or sound	19.37	1.04	19.45	1.05	19.67	0.80	19.64	0.60
Same–different	18.91	1.24	18.91	1.19	19.09	1.11	18.95	1.27
Word reading	39.29	0.94	39.45	0.89	39.62	0.70	39.69	0.67
Pseudoword reading	37.02	2.83	37.23	2.64	37.93	2.15	38.03	1.93
Grammatical structures	13.74	2.02	13.82	1.93	14.16	1.90	13.99	2.01
Punctuation marks	9.94	1.34	10.00	1.53	10.32	1.05	10.36	1.09
Sentence comprehension	15.47	0.82	15.54	0.76	15.75	0.52	15.57	0.71
Text comprehension	11.91	2.73	11.98	2.61	12.80	2.88	12.60	2.79
Oral comprehension	4.14	2.07	4.54	2.10	5.19	2.10	5.14	1.89

Note. M = mean; SD = standard deviation. Normative values are taken from the PROLEC-R manual for Spanish primary school students from 3rd to 6th Grade.
